# Is it safe to combine abdominoplasty and posterior vaginal repair in one surgical session?

**DOI:** 10.4103/0970-0358.44922

**Published:** 2008

**Authors:** Azzam S. M. Farroha, Hala S. Y. Hanna

**Affiliations:** 1 Plastic Surgery Section, Baghdad Health Department, Ministry of Health, Iraq; 2 Gynecology Section, Baghdad Health Department, Ministry of Health, Iraq

**Keywords:** Abdominoplasty, colpoperineorrhaphy, perineorrhaphy, posterior vaginal repair

## Abstract

Many multiparous women complain of protruded and pendulous abdomens and vaginal outlet relaxation which affect their sexual relationships with their male partners. This study included 47 patients who had these complaints. Some of these patients were working outside the homes and all were mothers of 2–5 children. Due of their home and job responsibilities, they did not have enough time or money for multiple surgeries in more than one session.

**Material and Methods:**

The age of these patients was 26–54 years and all patients had poor skin elasticity, pendulous excess subcutaneous fat and skin below the level of the anterior vulvar commissure, and a lax musculoaponeurotic anterior abdominal wall. Also, all patients had a relaxed vaginal outlet and 32 patients had rectocele. Careful perioperative assessment and management was done for each patient to ensure fitness for the long operation and to avoid complications. The combined surgical session consisted of two steps: abdominoplasty and posterior vaginal repair. All the patients were kept in the hospital for two days and they returned to their usual routines in the third week after surgery, and they resumed their sexual relationships with their male partners in the sixth week after surgery.

**Results:**

There were no serious complications and this approach was convenient for the patients and their families. The recovery time of the combined surgical session was the same as that of just abdominoplasty, and significantly less than the sum of the recovery periods if the two surgeries had been performed in two sessions. The cost of the combined surgical session was significantly less than doing the surgeries in two sessions. All the patients had significant improvement in their sexual relationships.

## INTRODUCTION

In the past, abdominoplasty has been combined safely and effectively with other elective surgeries such as liposuction, mammoplasty, abdominal hysterectomy, and cholecystectomy.[1–4] However, it has not been reported in combination with posterior vaginal repair (colpoperineorrhaphy or perineorrhaphy).

Many multiparous women complain of protruded and pendulous abdomens and relaxed vaginal outlets that affect their sexual relationships with their male partners. These combined complaints have been typically treated by performing abdominoplasty in one session and posterior vaginal repair in another session. As both these surgeries are considered of low priorities in public hospitals, patients usually undergo these surgeries in private hospitals. Performing these two operations in two different sessions costs the patients and their families a lot of money and time. Therefore, a solution for this problem was sought through team work between a plastic surgeon and a gynaecologist.

## MATERIALS AND METHODS

Between 2001 and 2006, 47 multiparous women underwent a combined abdominoplasty and posterior vaginal repair in one surgical session in Baghdad, Iraq.

The chief complaint of all patients and their male partners was difficulty in having sex and enjoying the sex as before, especially in the traditional position. All patients related this difficulty to the laxity in their vaginal outlets and to their pendulous and protruded abdomens. The history and physical examination of these 47 patients showed the following findings:

All 47 patients had male partners; 38 patients were housewives and nine patients were employed.

Thirty-nine patients had undergone only vaginal deliveries; six patients had undergone both vaginal deliveries and caesarean sections and two patients had undergone only caesarean sections.

Sixteen patients gave history of complications of episiotomy (wound infection and dehiscence).

All patients gained weight after each delivery and they had not performed pelvic or any other physical exercises properly. Forty -two patients were considered overweight and five patients were considered obese (but no one had massive obesity).

All patients complained of protruded and pendulous abdomens which interfered with intercourse. All patients had lax musculoaponeurotic structure in their anterior abdominal walls, poor skin elasticity, and huge excess of subcutaneous fat and skin that reached below the level of the anterior vulvar commissure in the standing position. None of the patients had transverse upper abdominal scar that might affect the abdominal flap viability.

Nine patients had hernias; two of them were lower paraumbilical hernias and the other seven were lower abdominal incisional hernias. The incisional hernias were at the scar of previous caesarean sections in three patients and scars of previous other laparotomies in four patients.

All patients complained of relaxation of the vaginal outlet (introital slackening), which interfered with sexual satisfaction; 32 of these patients also had rectocele.

Five patients had mild cystocoele but no one had symptomatic cystocoele or cystocoele that required conservative or surgical treatment. Three patients had a history of anterior vaginal repair at least two years ago.

All patients were checked for fitness for this combined surgical session. They did not have any conditions such as ischaemic heart disease, uncontrolled hypertension, diabetes, history of DVT, bleeding tendency, or massive obesity that might increase the possibility of complications and affect the safety of patient during the long major operation.

None of the male partners complained of disorders in erection or ejaculation. Seven of the male partners complained of protruded and pendulous abdomens but only two of them underwent abdominoplasty and liposuction at a later stage. None of the patients or their male partners complained of any further physical or psychological disorder which might affect their sexual relationships

All patients and their male partners were asked to stop smoking for one month before the surgery and at least two months after the surgery, to prevent the complication of anaesthesia, improve healing, and avoid coughing which might cause wound dehiscence. All patients were asked not to take aspirin for two weeks before the surgery in order to avoid massive bleeding and haematoma.

All patients were clearly informed that this combined surgical session would be conducted in two steps. The first step was to be used for the clean surgery (abdominoplasty) and the second step for the relatively contaminated surgery (posterior vaginal repair). Thus, if intraoperative complications happened during the first step, the second step would be cancelled.

When the patients were admitted into the hospital, the usual preoperative bowel, perineal, and vaginal preparations were performed.[5] Also, skin marking for abdominoplasty was performed; the postoperative pubic hair line was marked 5–7 cm above the anterior vulvar commissure.[2]

A prophylactic dose of heparin (5000 units) was given subcutaneously to five obese patients to avoid deep venous thrombosis (DVT); a second dose was given 12 hours after the first one.[6,7] A prophylactic dose of a 3^rd^ generation cephalosporin was given intravenously for all patients (no patient gave any history of sensitivity) and another two doses every 12 hours.[6]

A Foley's catheter was secured after induction of general anesthesia. The surgery started with the patients in a supine position after which the we had to semi-flex their hip to complete the abdominoplasty. At the end of the first step, the dressing was secured and the second part of the surgery was done in the lithotomy position.

In the first step of the operative session, all patients underwent a major abdominoplasty for repairing the lax muscles of the anterior abdominal wall, translocation of the umbilicus, and resection of the excess pendulous skin and fat.[2] Also, the abdominal hernias were repaired in nine patients.

In the second step of the surgical session, posterior colpoperineorrhaphy was performed on 32 patients to repair the pelvic floor muscles and posterior vaginal wall fascia, correct the rectocele, and reconstruct the perineal body.[5,8–11] Fifteen patients underwent only perineorrhaphy and levator plication.

All patients were kept in the hospital for two days. The Foley's catheter was removed on the morning after surgery and gradual ambulation was started. The patients had been instructed to rest at home for one week and to gradually increase their activities in the second week. Also, they had been instructed to eat healthy foods to avoid constipation. All patients returned to their usual work in the third postoperative week. All patients resumed sexual activity with their male partners in the sixth postoperative week. Follow-up of each patient was for at least for six months to assess the satisfaction of patients and their partners.

## RESULTS AND DISCUSSION

The majority of the patients were 41–45 years old [Table 1]. Multiparous women at this age have usually completed their families and do not plan to have more children. Also, these patients were fit for long major operations.

**Table 1:** Age distribution of patients and their children

*Age of the patients (years)*	*Number of patients*	*Number of children for each patient*
26–30	2	2–4
31–35	5	3–5
36–40	7	2–5
41–45	16	2–5
46–50	10	2–5
51–54	7	2–5

There were relatively fewer patients in the younger age groups and the number of patients increased with increasing age because multiparous women at these ages were still planning to have more children. Also, the effect of age was not very obvious in these groups, especially in the youngest group. The number of patients was relatively high in the older age groups, because the effect of age became more obvious (loss of skin elasticity and prolapsed genital organs), in addition to the effects of previous pregnancies and childbirths. However, with increasing age, the patients became less fit for long major operations.

The combined surgical session was safe.

- Abdominoplasty and posterior vaginal repair were done for all patients. There were no intraoperative complications that prevented the completion of both steps of the operative session.
- The mortality rate was zero.
- None of the 47 patients complained of any serious or major complications. Literature reports reveal an incidence of up to 16% for serious and major complications such as massive blood loss, large haematomas (which required surgical intervention), large seromas (which required aspiration or surgical drainage), cellulitis or abscesses (which required hospitalization and intravenous antibiotic treatment), DVT, pulmonary embolism, respiratory decompensation, and skin flap necrosis.[2,12,13] The absence of major and serious complications in this series was due to the very careful selection of patients; any patient with a risk for complications was not included in this combined operative session. Also, the careful preoperative preparation and postoperative monitoring and follow-up closely supervised by the consultant were contributing factors to the absence of complications.
- Some of the patients complained of minor and non serious complications of the abdominoplasty:
  One patient had a minor haematoma on the left side of the abdominal wound due to the blockage of the drain at that site. No intervention was required and spontaneous reabsorption occurred.
  Two patients had minor seromas of the abdominal wounds due to early restoration of activities in the second postoperative week without wearing corsets or binders. No treatment was required and spontaneous reabsorption occurred.
  One patient had a minor abdominal wound infection surrounding two stitches; it caused a wound dehiscence that was 3 cm long, which was treated conservatively.
  Four patients had bilateral ‘dog’ ears in the abdominal scar, which were corrected surgically under local anaesthesia about two months postoperatively.
- None of the 47 patients experienced any of the complications of the posterior vaginal repair such as haematoma, wound dehiscence, infection, and postoperative dyspareunia.[5]
- The combined surgical session was very convenient for the patients and their families.
- The cost of combining the abdominoplasty and the posterior vaginal repair in one session was about two thirds of the total cost of performing the two operations in two separate sessions. In the combined operative session, the major operation theatre and general anaesthesia were used once for each patient. The average time in the operation theatre required for the combined operative session was less than the sum of the lengths of time for abdominoplasty and posterior colpoperineorrhaphy in two separate sessions because there was only one time preparation of the patient and induction of general anaesthesia for the combined session. Each patient was admitted to the hospital just once and for two days only. All resources used during the preoperative preparation as well as follow-up were for one session.
- The recovery time required after combining abdominoplasty and posterior colpoperineorrhaphy was about six weeks, which is the same as doing abdominoplasty alone. It was significantly less than the total recovery time required after doing the two surgeries in two separate sessions.

All patients and their male partners experienced significant improvement in their sexual relationships. The level of improvement was assessed through asking each couple (the patient and her partner) to describe improvements as good to excellent, moderate, no or minimal improvements [Table 2]. The improvement in the sexual relationships was due to the improvement in sexual intercourse. Intercourse became less difficult (less effort and better penetration) because the abdominoplasty solved the problem of the protruding pendulous abdomens. Sexual satisfaction was improved because the colpoperineorrhaphy or perineorrhaphy tightened the vagina, causing better sensation during intercourse.

**Table 2:** Improvement in sexual intercourse at the third postoperative month

*Level of improvement in sexual satisfaction*				

	*none*	*mild*	*moderate*	*good*	*excellent*
No. of couples	0	0	7	16	24

Forty-four couples reported good to excellent improvement, seven couples reported moderate improvement, and no couples reported none or minimal improvements in their sexual relationships. The moderate improvement of seven couples was related to the protruded pendulous abdomens of their male partners. Two of these male partners underwent abdominoplasty and liposuction and later reported excellent improvement.

## CONCLUSIONS

Many multiparous women complain of problems in the sexual relationships with their male partners due to the effects of childbirth and age, leading to a combination of protruded and pendulous abdomens affecting intercourse performance and laxity of the vaginal outlet which affect sexual satisfaction.

The combined surgical session is safe for the patients but requires a careful selection of the patients, as well as careful preoperative preparation and postoperative monitoring and follow-up.

The combined surgical session is very convenient for the patients and their families because it achieves positive outcomes after a single operative session with one recovery period and less cost than doing the surgeries in multiple sessions.

It is always useful to remember that problems in the sexual relationships could be due to the female, male or both.

Teamwork between plastic surgeons and gynaecologists is very important to improve the quality of health care services provided for the patients.
